# Frequency of *SCA8*, *SCA10*, *SCA12*, *SCA36*, *FXTAS* and *C9orf72* repeat expansions in SCA patients negative for the most common SCA subtypes

**DOI:** 10.1186/s12883-017-1009-9

**Published:** 2018-01-09

**Authors:** Gülsah Aydin, Gabriele Dekomien, Sabine Hoffjan, Wanda Maria Gerding, Jörg T. Epplen, Larissa Arning

**Affiliations:** 1 Department of Human Genetics, Ruhr-University, Gebäude MA5/39, Universitätsstraße 150, 44801 Bochum, Germany; 2 Faculty of Health, University Witten-Herdecke, Alfred-Herrhausen-Strasse 50, 58448 Witten, Germany

**Keywords:** Spinocerebellar ataxia, Repeat expansions, SCA8, Fxtas, *C9orf72*

## Abstract

**Background:**

Spinocerebellar ataxia (SCA) subtypes are often caused by expansions in non-coding regions of genes like *SCA8*, *SCA10*, *SCA12* and *SCA36.* Other ataxias are known to be associated with repeat expansions such as fragile X-associated tremor ataxia syndrome (FXTAS) or expansions in the *C9orf72* gene*.* When no mutation has been identified in the aforementioned genes next-generation sequencing (NGS)-based diagnostics may also be applied. In order to define an optimal diagnostic strategy, more information about the frequency and phenotypic characteristics of rare repeat expansion disorders associated with ataxia should be at hand.

**Methods:**

We analyzed a consecutive cohort of 440 German unrelated patients with symptoms of cerebellar ataxia, dysarthria and other unspecific symptoms who were referred to our center for SCA diagnostics. They showed alleles in the normal range for the most common SCA subtypes SCA1-3, SCA6, SCA7 and SCA17. These patients were screened for expansions causing SCA8, SCA10, SCA12, SCA36 and FXTAS as well as for the pathogenic hexanucleotide repeat in the *C9orf72* gene.

**Results:**

Expanded repeats for SCA10, SCA12 or SCA36 were not identified in the analyzed patients. Five patients showed expanded SCA8 CTA/CTG alleles with 92-129 repeats. One 51-year-old male with unclear dementia symptoms was diagnosed with a large GGGGCC repeat expansion in *C9orf72*. The analysis of the fragile X mental retardation 1 gene (*FMR1*) revealed one patient with a premutation (>50 CGG repeats) and seven patients with alleles in the grey zone (41 to 54 CGG repeats).

**Conclusions:**

Altogether five patients showed 92 or more SCA8 CTA/CTG combined repeats. Our results support the assumption that smaller *FMR1* gene expansions could be associated with the risk of developing neurological signs. The results do not support genetic testing for *C9orf72* expansion in ataxia patients.

## Background

Autosomal dominant and autosomal recessive inheritance is responsible for the majority of hereditary ataxia subtypes. The spinocerebellar ataxias (SCAs) are autosomal dominantly inherited with a worldwide distribution and an overall prevalence varying from 0.3 to 4.2/100000 [1]. Yet, epidemiological studies that have been conducted on these disorders are relatively rare, and the prevalence estimations vary considerably between countries. Leading symptoms of SCA are cerebellar dysfunctions such as uncoordinated limb movements, unsteady gait, loss of coordination, dysarthria and disturbance of oculomotor control [2]. However, a great variability in the severity and type of symptoms is observed, and the age of onset as well as the rate of disease progression varies between individuals [3]. Up to now, about 35 SCA loci have been mapped and 22 genes have been identified [4]. However, this number is expected to increase in the future, since whole genome or exome sequencing by next-generation sequencing (NGS) offers great potential for finding causative genes of rare subtypes [5]. The high complexity of the clinical picture and the variable genetic background make accurate differential diagnosis often difficult. The most frequent SCA forms are the polyglutamine expansion SCAs that are caused by expanded CAG trinucleotide repeats encoding polyglutamine tracts in various genes [6]. In these cases the repeat expansions can be easily and cost-effectively tested using standard PCR methods. In routine diagnostics the CAG fragment lengths for SCA1, SCA2, SCA3, SCA6, SCA7 and SCA17 are often tested in series. SCA forms that are caused by rare conventional mutations in SCA genes can be tested by Sanger-based DNA sequencing, long time the gold standard for mutation detection [6]. The diagnostic procedure of this gene-by-gene sequencing is cost- and time-consuming, and part of the ataxia patients remain thus without molecular genetic diagnosis. This fact renders difficult to predict a patient’s disease progression, to offer effective genetic family counselling and to identify potential novel therapies. Therefore, targeted NGS approaches (gene panels) as well as next-generation whole exome sequencing are becoming more widespread in routine molecular diagnostics for patients with ataxia [7]. However, since NGS at present is not suitable for detecting (trinucleotide) repeat expansions [8], a pre-NGS testing for common polyglutamine expansion SCAs appears mandatory. Further, SCA subtypes caused by expansions in non-coding regions of genes responsible for SCA8, SCA10, SCA12 and SCA36 as well as other ataxias known to be associated with repeat expansions such as fragile X-associated tremor ataxia syndrome (FXTAS) should be taken into account before applying NGS-based diagnostics.

In order to define an optimal diagnostic strategy, more information about the frequency and phenotypic characteristics of rare repeat expansion disorders associated with ataxia would be helpful [9, 10]. We therefore analyzed a cohort of unrelated German 440 patients with symptoms of cerebellar ataxia, dysarthria and other unspecific symptoms who showed alleles in the normal range for the routinely tested SCA types. After screening for expansions in SCA8, SCA10, SCA12, SCA36 and FXTAS, the cohort was additionally evaluated for a pathogenic hexanucleotide repeat in the *C9orf2* gene, which has recently been identified as a common pathogenic mutation in families with autosomal dominant frontotemporal dementia (FTD) and amyotrophic lateral sclerosis (ALS) [11]. However, the spectrum of neurological conditions associated with the repeat expansion in *C9orf72* is very broad, including rarely cerebellar ataxias [12]. In order to check if *C9orf72* expansions also contribute to the spectrum of neurological conditions found in our cohort, we included the screening for *C9orf72* expansions in our analyses.

## Methods

### Patients

We studied a consecutive series of 440 unrelated German patients who were referred to the Department of Human Genetics in Bochum for genetic testing for SCA in the years 2008-2015. They show alleles in the normal range for SCA1, SCA2, SCA3, SCA6, SCA7, and SCA17. SCA diagnosis was requested through clinical neurologists or other specialists. The cohort is clinically heterogeneous, with mild to severe symptoms of cerebellar ataxia, dysarthria and other neurologic symptoms of varying duration. Clinical data and family history were reviewed to the extent available. The study methods were approved by the institutional review board of the Ruhr-University Bochum. Written informed consent for genetic studies was obtained from all patients enrolled in the study.

### Molecular testing

Genomic DNA was extracted from peripheral blood leukocytes using the conventional method. For SCA8, SCA10 and SCA36 triplet repeat primed PCR (TP-PCR) assays based on the method of Warner et al. were performed in order to detect very large expansions that cannot be amplified by PCR-fragment analyses using primer pairs flanking the respective repeat [13]. Patients that appeared homozygous for one allele in the normal fragment analyses were reanalyzed with TP-PCR in order to detect very long pathogenic repeats that could have been missed by conventional PCR [14–16]. In order to analyze the GC-rich *C9orf72* hexanucleotide repeat expansions, we applied a flanking and repeat-primed PCR assay according to Cleary et al. [17]. Fragile X mutations were analyzed by conventional fluorescent PCR which is sufficient to detect normal and premutation alleles and therefore to exclude the diagnosis of Fragile X syndrome (FXS) due to full mutations. PCR products were separated and analyzed on an ABI 3500XL Genetic Analyzer (Applied Biosystems) and evaluated using the GeneMapper v4.1 software (Applied Biosystems). All primer sequences are available upon request.

## Results

### SCA8

The majority of patients showed SCA8 CTA/CTG combined repeat lengths between 15 and 42. Repeats of 92 or longer were observed in five patients (92-129, Table 1). Patients that appeared homozygous for one allele in the normal fragment range were reanalyzed with fluorescent TP-PCR in order to detect very long pathogenic CTA/CTG repeat blocks [14]. None showed a pathogenic expansion.

**Table 1:** Genetic and clinical features as provided from the referring physician of the five patients (two females/ three males) with SCA8 repeat expansions

Patient	(CTA/CTG)n allele 1/2		Symptoms	Age at onset	Family history
1	22	129	Gait ataxia, dysarthria, atrophy of the cerebellum	19-21	affected mother
2	26	125	Atrophy of the cerebellum	NA	NA
3	24	122	Gait ataxia, dysarthria, saccadic eye movements	19-21	no
4	30	116	Stance and gait ataxia, dysarthria, saccadic eye movements	45-47	no
5	25	92	Movement disorder, dementia	41-43	no

*NA* not available

### SCA10, SCA12, SCA36

No expanded repeats for SCA10, SCA12 or SCA36 were found in the 440 patients. For SCA10 the number of ATTCT pentanucleotide motifs ranged from 12 to 25. The most common normal alleles in our German cohort contained 15 or 16 ATTCT repeats. Patients that appeared homozygous were reanalyzed with fluorescent repeat-primed PCR. Normal SCA12 alleles ranged from 9 to 24 CAG repeats, with 10 CAG repeats being most frequent (60%). For SCA36 the fragment analysis revealed that normal repeats ranged from 5 to 13 GGCCTG repeat units. Extreme GGCCTG repeat expansions were excluded by repeat-primed PCR analysis.

### Fxtas

Analysis of the fragile X mental retardation 1 gene (*FMR1*) identified one patient with a premutation above 50 CGG repeats. This female patient with 57 repeats presented at the age of 57 years with slowly progressive stance and gait ataxia and dysarthria. Additionally, seven patients (3 males/4 females) were identified with alleles in the grey zone, ranging in size from 41 to 54 CGG repeats (Table 2).

**Table 2:** Genetic and clinical features as provided from the referring physician of the eight patients (five females/three males) with *FMR1* premutation/alleles in the grey zone

Patient	(CGG)n allele 1/2		Symptoms	Age at onset	Family history
I	30	57	Slowly progressive stance and gait ataxia and dysarthria	56-58	no
II	53		Stance and gait ataxia	NA	no
III	30	49	Unsteady gait with a tendency to fall	NA	no
IV	31	47	Cerebellar ataxia	NA	affected brother
V	30	45	Gait ataxia, dysarthria, nystagmus, diagnosed with fibromyalgia	59-61	no
VI	41		Progressive gait disturbance, erectile dysfunction, micturition disturbance	NA	no
VII	41		Unsteady gait, coordination disturbances of the hands, saccadic eye movements, mild dysarthria	49-51	no
VIII	32	41	Cerebellar ataxia	NA	no

*NA* not available

### C9orf72

**Fig. 1:**
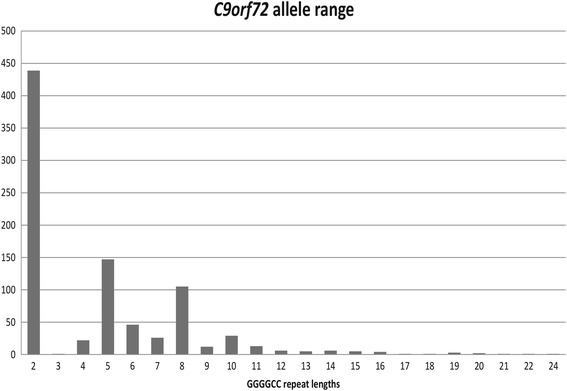
Distribution of the GGGGCC repeat lengths in the non-expansion carriers

The numbers of GGGGCC repeats varied from 2 to 24 units in the normal range (Fig. 1). Analyzing all apparently homozygous patients with fluorescent repeat-primed PCR identified one *C9orf72* mutation carrier in our cohort. The patient first appeared as homozygous for a short allele (2 GGGGCC repeat) using the routine assay, but reanalysis with fluorescent TP-PCR showed the typical pattern of an expansion as observed in the positive control.

## Discussion

In a first line genetic screening for SCA1, SCA2, SCA3, SCA6, SCA7 and SCA17 mutations 440 ataxia patients were tested negative, as requested through clinical neurologists or other specialists. These unrelated ataxia patients were subsequently assayed for repeat expansions in SCA8, SCA10, SCA12, SCA36, FXTAS and *C9orf72*.

### SCA10, SCA12, SCA36

No expanded repeats for SCA10, SCA12 or SCA36 were found in this cohort. SCA10 mutations are unstable expansions of a pentanucleotide (ATTCT) repeat in intron 9 of the SCA10 gene. In patients with SCA10 very large expansions of 800 to 4500 ATTCT repeats are found; normal alleles comprise 10 to 22 repeats [18, 19]. In our German cohort the number of ATTCT pentanucleotide motifs ranges from 12 to 25, with alleles of 15 or 16 ATTCT repeats being most frequent. In support of a founder mutation in the Mexican population, our results substantiate the assumption that SCA10 is a rare cause of ataxia in ethnic populations other than Latin American [6, 18, 19]. SCA12 is associated with a CAG expansion upstream of the transcription start site of the *PPP2R2B* gene, encoding a brain-specific regulatory subunit of the protein phosphatase PP2A. CAG repeats in healthy controls range between 7 and 28, and expansions between 46 and 78 triplets have been associated with the disease so far [20]. The unexpanded SCA12 alleles in our cohort ranged from 9 to 24 CAG repeats, with 10 CAG repeats being most frequent in accordance with previous reports [21]. There are similar results for SCA36, which is caused by a large (>650) expansion of an intronic GGCCTG repeat in the *NOP56* gene [22]. At present, affected families have predominantly been described in Japan, Spain, and France, while no expansions were found in another German cohort [16, 22–24]. In healthy controls the hexanucleotide repeat ranges between 3 and 14 repeat units, with the nine-repeat allele being the most frequent one in Europeans [16, 22–24]. In our cohort the detected GGCCTG repeat alleles ranged between 5 and 13 units, also with the nine-repeat allele being most frequent, while no sample showed a pathogenic pattern.

### SCA8

In SCA8 analysis, expanded lengths are found comparatively frequently for the combined CTA/CTG combined repeat. In 1999 the expansion of an untranslated CTG repeat on chromosome 13q21 was proposed to cause SCA8 [25]. The polymorphic CTG repeat is situated in direct vicinity to a polymorphic CTA sequence (1-21 repeats) whose length is usually quoted in combination with the CTG repeat length. The potentially pathogenic SCA8 alleles associated with symptoms range from 68 to over 300 combined (CTA)n/(CTG)n repeats [26]. In healthy controls more than 99% show repeat lengths between 16 and 37 combined repeats [25]. However, because expanded alleles were also detected among healthy controls and expansion did not co-segregate with disease in several families, the established SCA8 pathogenic threshold is questionable [26–28]. Common initial symptoms of SCA8 are scanning dysarthria with gait instability with disease onset typically occurring in adulthood [29]. We also found a relatively high frequency of expanded SCA8 repeats in our cohort of patients. Altogether five patients (1.14%) showed repeat sizes of 92 or more combined repeats. The symptoms of the corresponding patients comprise various neurological symptoms (Table 1). Their family histories are almost consistently negative. This is not too surprising when considering, that because of the reduced penetrance of the repeat expansion in SCA8, the most common presentation is a single affected person in a family [29]. Together with reports on the presence of pathogenic repeat lengths in healthy control cohorts and in patients with other identified genetic causes for ataxia, the reduced penetrance led to the assumption that the expanded CTG repeat may be a rare polymorphism which is in linkage disequilibrium with other mutations at the locus associated with SCA8 and/or other factors contribute to the SCA8 phenotype leading to reduced or incomplete penetrance [29]. In this situation, diagnostic testing for SCA8 should be considered when the family history suggests that the symptoms are sporadic or inherited in an autosomal recessive manner. However, diagnostic testing results for SCA8 should be interpreted with caution, especially when used for genetic counseling.

### Fxtas

In 2001 Hageman et al. described five elderly men with a trinucleotide CGG repeat expansion in the premutation range (55 to 200 CGG repeats) in the *FMR1* gene. These patients showed late-onset neurological symptoms consisting of progressive action tremor associated with executive function deficits and generalized brain atrophy [30]. Expansions over 200 CGG repeats located in the promoter region of the *FMR1* gene (full mutation) generally lead to transcriptional silencing and result in FXS [31]. Expansions in the premutation range are, however, associated with an increased mRNA transcription [32]. FXTAS classically presents with kinetic tremor and cerebellar gait ataxia in elderly male *FMR1* premutation carriers, and also female premutation carriers with a milder form of FXTAS have been reported [33, 34]. However, in recent years it became apparent that the FXS associated premutation disorders correlate with a broad spectrum of symptoms that go beyond the clinical picture of FXTAS and the fragile X-associated primary ovarian insufficiency (FXPOI) [35, 36]. Clinical symptoms also include peripheral neuropathy, autonomic dysfunction as well as progressive cognitive decline and diverse neuropsychological problems such as parkinsonism [36]. Because of the heterogeneity of neurological phenotypes in FXTAS, patients are often initially diagnosed with other disorders like SCA or Parkinson disease (PD), suggesting that FXTAS might explain a substantial number of apparently sporadic cases of late-onset ataxia. Previous studies showed that the frequency of FXTAS in patients with movement disorders ranges from 0 to 5% in different populations [34, 37–41]. Previous published reports of FXTAS have also suggested that woman are far less frequently affected than males, possibly due to the presence of the second X chromosome and random X inactivation and/or a sex-specific protective effect, perhaps related to estrogen [33].

Smaller *FMR1* gene expansions with 45-54 CGG repeats have been designated as gray zone or intermediate alleles due to their lower risk of expansion into a full mutation causing FXS in later generations [42, 43]. However, the exact boundaries for the intermediate range vary from 34 to 60 CGG repeats [44]. Interestingly, gray zone alleles also show significantly increased transcriptional activity as compared to that observed in common alleles up to 39 CGG [44]. These alleles are associated with various neurological phenotypes, including classical features of PD, with most patients showing asymmetric rest tremor, bradykinesia, and rigidity, but also kinetic tremor and mild gait ataxia or anxiety [45–47]. Therefore, our results add to the growing body of evidence that gray zone alleles are associated with neurological symptoms. It is also interesting to note that we found a higher share of women than men amongst the patients with *FMR1* premutations or grey zone alleles. Possibly women are underdiagnosed in the current diagnostic practice for FXTAS, especially when they present with a clinical course that is typical of males with FXTAS.

### C9orf72

Large expansions of a GGGGCC repeat (>400 repeats) located in intron 1 of the *C9orf72* gene are associated with familial ALS and FTD [48]. The frequency of the *C9orf72* expansion in ALS patients varies. It is remarkably high in Finland and some other regions and infrequent in Asian populations, an observation that supports the theory of a common founder effect [49, 50]. Over 90% of the European population show variable *C9orf72* repeat lengths between two and ten repeat units [48]. This pattern of distribution was also shown in our cohort (Fig. 1). Besides the strong link to ALS and FTD, which are both heterogeneous diseases, the phenotypic spectrum of *C9orf72* expansions extends to other neurodegenerative syndromes such as PD, progressive muscular atrophy (PMA), primary lateral sclerosis (PLS), Huntington-like disease as well as ataxia syndromes [51–54]. The patient diagnosed with a large GGGGCC repeat expansion in our cohort was a 51-year-old man who is described as having unclear dementia syndromes, neurological restrictions and putative early psychiatric problems. However, no ataxia was mentioned. There was a dominant family history of a similar syndrome in his father. Since dementia is the dominant symptom in this patient, this case does not broaden the phenotypic spectrum of pathogenic *C9orf72* repeat expansions, but it underlines the importance that special attention should be given to patients with dementia referred for molecular diagnostic of various neurodegenerative disorders.

Although our study has multiple strengths, there are also limitations to consider. First, this is a retrospective study; therefore patient data are partially incomplete like history of symptoms, age at onset, family history and exclusion criteria like positive history of alcohol abuse. Another limitation of this study is that there were no healthy controls included.

## Conclusion

Concerning the implications for genetic SCA testing, one can conclude from these data that testing for SCA10, 12 and 36 is not absolutely essential in a German patient cohort. Expanded SCA 8 repeats occur quite frequently, but with regard to its reduced or incomplete penetrance it is difficult to reliably assess the potential consequences of SCA8 expansions on the phenotype. Therefore, it should be critically considered whether this diagnostic should be a fixed component of SCA routine diagnostics. A similar situation exists in evaluation of the *FMR1* gray zone or intermediate alleles. Our results support the assumption that smaller *FMR1* gene expansions are also associated with the risk of developing neurological signs, in particular also female patients should be considered in the diagnostic practice for FXTAS. Finally, our study does not support genetic testing for *C9orf72* expansion in ataxia patients. Further efforts on this field of research are required.

## Abbreviations

ALS: Amyotrophic lateral sclerosis
DM1: Myotonic dystrophy type 1
DNA: Deoxyribonucleic Acid
FTD: Frontotemporal dementia
FXS: Fragile X syndrome
FXTAS: Fragile X-associated tremor ataxia syndrome
HD: Huntington disease
NGS: Next-generation sequencing
ORFs: Open reading frames
PLS: Primary lateral sclerosis
PMA: Progressive muscular atrophy
RNA: Ribonucleic acid
SCA: Spinocerebellar ataxia
TP-PCR: Triplet repeat primed polymerase chain reaction
